# Digital Twins as catalysts for Whole Person Health Mind Body Medicine in Integrative Oncology

**DOI:** 10.3389/fonc.2026.1868314

**Published:** 2026-06-08

**Authors:** Claudia M. Witt

**Affiliations:** 1 Complementary and Integrative Digital Health Institute of Primary Care, University of Zurich and University Hospital Zurich, Zurich, Switzerland; 2 Digital Society Initiative, University of Zurich, Zurich, Switzerland; 3 Comprehensive Cancer Center Zurich (CCCZ), Clinical Program, Zurich, Switzerland

**Keywords:** AI, artificial intelligence, Digital Twin, integrative oncology, Mind Body Medicine, Whole Person Health

## Abstract

Artificial intelligence and digital health technologies are reshaping oncology through increasingly personalized, predictive, and data-driven care. Among these innovations, medical Digital Twins - dynamic virtual software representations of individual patients - are emerging as tools for simulating disease trajectories, forecasting treatment responses, and supporting clinical decision-making. To date, most oncology focused Digital Twin research centers on tumor biology, imaging, genomics, radiotherapy optimization, and treatment response. Integrative Oncology requires a broader perspective. As a patient-centered field that emphasizes Whole Person Health across prevention, treatment, rehabilitation, survivorship, and long-term health promotion, Integrative Oncology depends on biological, behavioral, psychosocial, environmental, and lifestyle-related data that remain underrepresented in digital health infrastructures. This paper argues that Digital Twins could advance Whole Person Health in Integrative Oncology if they are designed to integrate not only disease- and treatment-related data but also key domains of Mind Body Medicine, including physical activity, diet, sleep, stress regulation, self-care, social and other relationships, living conditions, and personal preferences. Drawing on a SWOT analysis and a Whole Person Health Mind Body Medicine model, the paper identifies strengths and opportunities, including personalization, longitudinal monitoring, treatment coordination, and adaptive self-care support. It also highlights challenges related to data quality, interoperability, digital literacy, privacy, autonomy, equity, and reductionist interpretations of health. The central challenge is therefore not only technological but preparatory. Integrative Oncology must actively contribute to interoperable data standards, ethical governance, clinical validation, and interdisciplinary collaboration to ensure that future Digital Twins support, rather than marginalize, whole person, human-centered cancer care.

## 1 Digital Twins

Artificial intelligence (AI) and digital health technologies are rapidly transforming oncology, with recent advances in machine learning, multimodal data integration, and real-time monitoring reshaping cancer care. Among emerging innovations, medical Digital Twins represent a particularly ambitious concept. They are dynamic, data-driven virtual representations of individual patients, capable of simulating disease trajectories and treatment responses. By enabling continuous data integration and interpretation, they augment clinical decision-making. These “patients-in-silico” allow visualization of disease states, prediction of progression, and simulation of treatment scenarios. Compared with conventional approaches, Digital Twins offer a more precise and individualized framework, moving beyond generalized treatment strategies toward truly personalized healthcare (1).

Recent literature emphasizes that Digital Twins are not yet mature clinical tools, but rather emerging systems expected to evolve over the next decade into clinically actionable platforms. Current implementations remain largely organ-specific and lack integration across biological, behavioral, and environmental domains.

### 1.1 Digital Twins in oncology

Digital Twins in oncology are evolving from mechanistic tumor models toward increasingly complex, data-driven systems. Early multiscale modeling demonstrated the feasibility of simulating tumor growth and treatment response, while more recent approaches have integrated imaging, genomic, and clinical data to predict disease trajectories. Emerging evidence indicates that Digital Twins can capture tumor heterogeneity and support personalized treatment strategies. In radiotherapy, patient-specific Digital Twin models have enabled optimization of dosing, improving tumor control while reducing toxicity (2). Similarly, AI-driven Digital Twin frameworks in head and neck cancer have demonstrated improvements in survival outcomes and reductions in treatment-related morbidity (2). In lung cancer, models combining imaging and machine learning achieve high predictive accuracy for classification and clinical forecasting, while large-scale initiatives such as PRIMAGE (3) highlight the potential of integrating imaging biomarkers and clinical data for precise tumor characterization.

Despite these advances, current applications remain largely focused on tumor biology and treatment optimization. The integration of behavioral, lifestyle, and psychosocial factors—central to Integrative Oncology - is still limited, reflecting gaps in data availability and interoperability. Recent reviews emphasize that Digital Twins are increasingly capable of modeling physiological processes across scales, from molecular interactions to organ-level dynamics, thereby enabling precision medicine (4). However, extending these models to Whole Person Health remains a key challenge for future development. No holistic Digital Twin integrating biological, behavioral, psychosocial, and environmental data into a single patient model currently exists. Present implementations are organ- or system-specific: a cardiac Digital Twin models hemodynamics (5), a tumor Digital Twin models tumor growth (6), but neither incorporates sleep, stress, social context, or lifestyle. Achieving such integration is a longer-term aspiration that will require interdisciplinary collaboration that Integrative Oncology is well positioned to help build.

## 2 Integrative oncology

Integrative Oncology provides a compelling framework for evaluating the development of Digital Twins in future medicine. It is defined as follows: “Integrative oncology aims to optimize health, quality of life, and clinical outcomes across the cancer care continuum and to empower people to prevent cancer and become active participants before, during, and beyond cancer treatment” (7). Its definition emphasizes patient-centered, individualized, and whole person care across the cancer continuum. At the same time, Integrative Oncology includes domains such as lifestyle, mind body practices, and complementary therapies, which have been historically underrepresented in biomedical data infrastructures.

In a data-driven future, a critical insight emerging from recent work is that data availability, not only technology, will determine the scope and inclusivity of Digital Twins. As highlighted in the integrative medicine AI perspective, “data are the lifeblood of AI systems,” and the absence of data for specific interventions directly leads to their exclusion from algorithmic recommendations (8). This has profound implications for Integrative Oncology. If data on interventions such as acupuncture, mind body practices, or lifestyle modifications are not systematically collected and integrated, these approaches risk becoming invisible within future Digital Twin-driven care models. This creates a critical tension: Digital Twins have the potential to operationalize Whole Person Health, yet they may also reinforce existing biases if Integrative Oncology data are not adequately represented. Recognizing and addressing this tension is essential for shaping the future of Integrative Oncology within a data-driven healthcare system.

## 3 Mind Body Medicine for Whole Person Health

Mind Body Medicine, as implemented in Integrative Oncology in Switzerland (9), is “a modern, science-based, integrative approach. It connects the body and mind, promotes self-care, and is used both for prevention and treatment. Through multimodal therapy approaches, it aims to reduce symptoms, strengthen personal resources, and enhance self-efficacy” (10).

**Figure 1:**
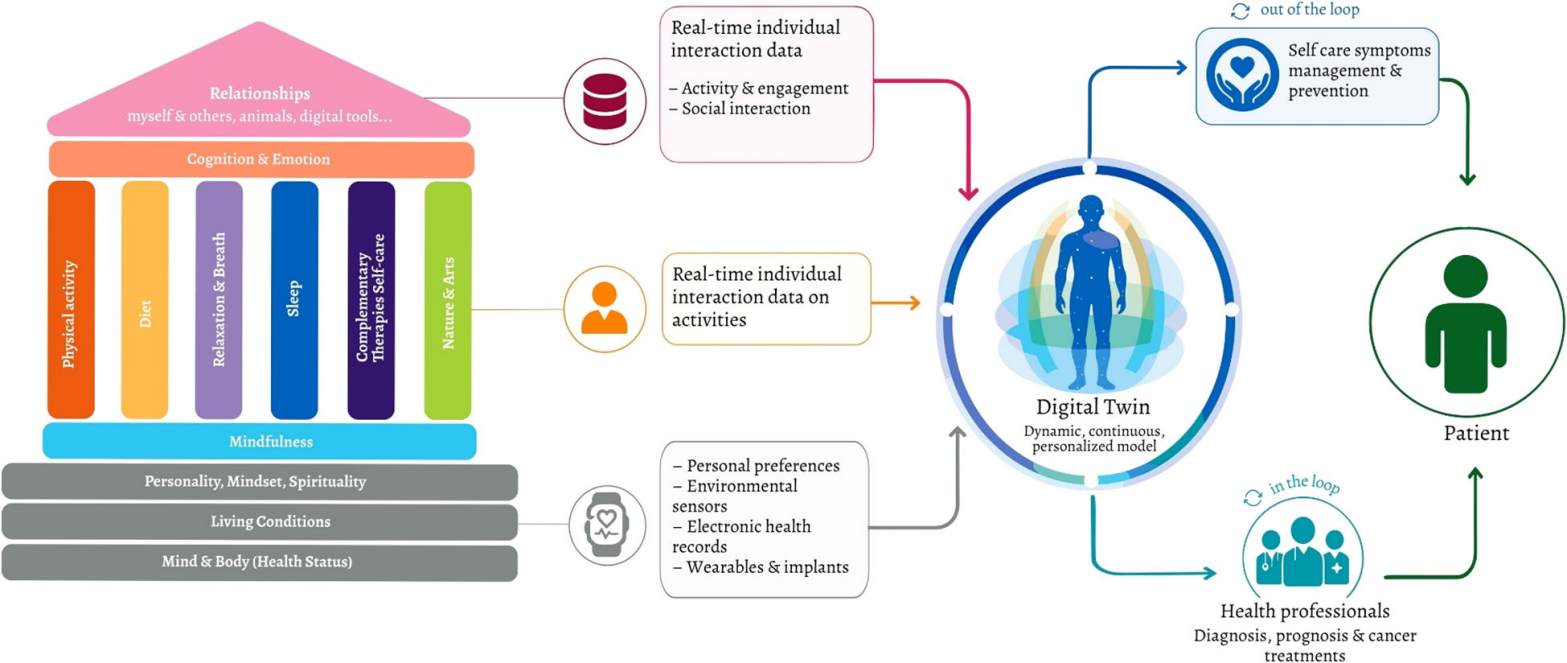
Digital Twins and Whole Person Health Mind Body Medicine for future integrative oncology.

Mind Body Medicine serves as a valuable bridge between Integrative Oncology and Digital Twin approaches, as it explicitly addresses, in both assessment and intervention, the interactions between biological, psychological, behavioral, social, and environmental determinants of health. This aligns with Whole Person Health, which “involves looking at the whole person—not just separate organs or body systems—and considering multiple factors that promote either health or disease” (11). The “Mind Body Medicine Whole Person Health Temple” (12) model (see **Figure 1**) depicts a multilayered structure. The foundation includes mind and body health status, living conditions, personality, mindset, and spirituality. Mindfulness functions as a central supporting layer, while six modifiable pillars represent key resources and interventions: physical activity, diet, relaxation and breath, sleep, complementary medicine self-care, and nature and arts. Cognition and emotion are positioned as higher-order processes influencing behavior, while relationships form the overarching level, including relationships with oneself, others, animals, nature, and digital tools.

This model provides a strong example for Integrative Oncology, as it shifts supportive care beyond isolated symptom control and toward a structured whole person framework. Mind Body Medicine therefore provides both a clinical content model and a patient-centered value framework for Digital Twins in Integrative Oncology.

## 4 Digital Twins as enablers of Whole Person Health

As summarized in the SWOT analysis in **Table 1**, Digital Twins could become an enabling framework for Whole Person Health in Integrative Oncology by combining biological, behavioral, psychosocial, environmental, and clinical information into a dynamic, personalized model of the patient. As illustrated in **Figure 1**, such a model could integrate real-time data from e.g. wearables, body implants, electronic health records, environmental sensors, personal preferences, activity patterns, engagement, and social interaction. This is particularly relevant for Mind Body Medicine, where core resources and therapeutic domains include physical activity, diet, sleep, relaxation and breath, complementary self-care, mindfulness, cognition and emotion, nature and arts, living conditions, and relationships. By incorporating these dimensions, Digital Twins could help shift supportive oncology care from static lifestyle advice toward adaptive, patient-centered strategies that respond to changes in symptoms, needs, goals, and context over time.

**Table 1:** Analyses on strength, weaknesses, opportunities and threats (SWOT) of Digital Twins in Integrative Oncology (8, 15).

**Strengths** Integrates multimodal oncology, sensor, lifestyle, and patient-reported data into a patient-specific model for prevention, diagnosis, prognosis, and therapy. Can simulate health trajectories and support risk forecasting, side-effect monitoring, and treatment coordination in cancer care. Aligns with patient-centered integrative medicine: AI tools can individualize acupuncture, support education, and enhance patient engagement. Provides a shared decision-support layer for interprofessional teams rather than replacing clinician judgment.	**Weaknesses** Digital-Twin performance depends on high-quality standardized data; current integrative/acupuncture records remain heterogeneous and may rely on free text or paper. Key acupuncture variables, including point codes/location, needle depth, retention time, stimulation parameters, outcomes, and PROMs, are inconsistently documented. Limited digital literacy among clinicians, patients, and researchers may slow safe implementation. Available digital-Twin governance findings are partly scenario-based and Swiss-centered, so transfer to other oncology systems requires caution.
**Opportunities** Supports precision integrative oncology through coordinated treatments, early risk detection, disease-course prediction, and individualized supportive care. Interoperable data standards, templates, and open-data principles could convert routine integrative oncology care into research-ready real-world evidence. Can extend care through remote monitoring, telemedicine, follow-up, and decentralized studies for patients with symptom burden or access barriers. Public hospital, university, and state-backed governance may strengthen trust, infrastructure, and equitable access.	**Threats** Mandatory or insurer-incentivized use could threaten autonomy, the right not to know, and continued access to non-digital care. Privacy and data-security failures, opaque algorithms, and commercial control could erode trust. Digital divides may amplify inequities if patients lack access, skills, or culturally appropriate support. Misinformation, automation pressure, external oversight, and possible displacement of clinician roles could weaken human-centered integrative care.

Yet translating social and contextual determinants of health, such as social support, housing, financial security, and cultural background, into computable Digital Twin inputs remain a technically and conceptually demanding challenge. These factors are dynamic and relational in ways that resist reduction to numerical values. Patient-reported screening tools offer one path toward their longitudinal capture alongside clinical and wearable data, with social determinants treated as time-varying inputs that can be updated as circumstances shift across the cancer trajectory. How much influence a reported social stressor should carry within a predictive model is not a purely technical decision, and patients themselves should be able to adjust these priorities in line with their current values and life context.

Realizing this potential depends on the responsible use of multimodal data, interprofessional collaboration, and clear boundaries between patient directed self-care and clinician guided cancer care. These conditions correspond directly to the opportunities and threats identified in the SWOT analysis (**Table 1**). Accordingly, Digital Twins should support self-care, symptom management, prevention, and survivorship while keeping diagnosis, prognosis, and cancer treatment decisions within health professionals oversight. This approach preserves the strengths and opportunities of Digital Twins (incl. personalization, continuity, treatment coordination, and earlier risk detection), while the weaknesses and threats identified in **Table 1**, namely data quality, privacy, autonomy, equity, digital literacy, and the risk of reducing Whole Person Health to optimization metrics, make clear why responsible design and governance are preconditions for realizing this potential.

## 5 Challenges beyond data availability and technology

The SWOT analysis in **Table 1** also highlights that the challenges associated with Digital Twins extend beyond data availability and technical implementation. Clinical validation remains a major requirement, particularly for whole system Digital Twin models that combine biological, behavioral, lifestyle, and treatment response data. Current Digital Twin developments are still based on single systems or narrowly defined datasets, and robust prospective evidence is needed before Digital Twins can be responsibly integrated into oncology care. In Integrative Oncology, this challenge is especially relevant, as interventions are often multimodal, individualized, and dependent on patient-reported outcomes, making standardized documentation and evaluation essential.

Ethical and social concerns are equally important. Systems that rely on continuous data collection amplify issues related to privacy, consent, data ownership, algorithmic bias, and transparency. There is also a risk that Digital Twins could promote an overly reductionist or optimization driven understanding of health, which may conflict with the humanistic and patient-centered values of Integrative Oncology. Concerns about autonomy, the right not to know, and pressure to follow digitally generated recommendations are therefore central threats. Equity must also be considered. Differences in digital literacy, access to technologies, and trust in digital health systems could widen existing disparities, including among patients who use integrative medicine (13). For these reasons, Digital Twins in Integrative Oncology should never be mandatory systems that replace clinician judgment or patient choice.

## 6 Preparing for the future

As Digital Twins will move from experimental models toward potential clinical application in the future, Integrative Oncology should prepare now to ensure that Whole Person Health is represented in future digital health systems. A first priority is the systematic generation and standardization (8) of data relevant to Integrative Oncology, including lifestyle factors, mind body interventions and other complementary therapies, patient-reported outcomes, symptoms, adverse events, and contextual factors such as preferences, social relationships, and living conditions. These data should be captured using interoperable terminologies, structured documentation templates, harmonized reporting standards, and metadata frameworks that allow integration with oncology records, wearable devices, environmental sensors, and other digital health sources.

Preparation also requires expanding existing professional Integrative Oncology competencies (14). Integrative Oncology health professionals will need sufficient digital literacy to understand how Digital Twins operate, what types of data they use, how their outputs should be interpreted, and where their limitations lie. This includes the ability to critically evaluate algorithmic recommendations, identify potential bias, communicate uncertainty to patients, and preserve shared decision-making. Ethical and governance competencies will be equally important, particularly in relation to consent, data ownership, privacy, transparency, equity, and the patient’s right to decline digital monitoring or digitally guided care.

Finally, Digital Twins should not be developed as purely technical tools. Their responsible implementation will require close collaboration among oncologists, integrative medicine practitioners, behavioral scientists, data scientists, engineers, ethicists, patients, and regulators. Integrative Oncology health professionals must be actively involved in these collaborations to ensure that future Digital Twins reflect the values of Whole Person Health, patient empowerment, and human-centered care. Most importantly, Digital Twins should be designed to support rather than replace the therapeutic relationship, clinical judgment, and patient choice.

## 7 Conclusion

Digital Twins represent a promising but still emerging paradigm for Integrative Oncology. Their potential lies in the ability to combine multimodal data, simulate individual disease trajectories, support treatment coordination, and enable more personalized decision-making. For Integrative Oncology, this potential is particularly relevant because Digital Twins could extend precision oncology beyond tumor-directed care and incorporate the broader determinants of Whole Person Health, including symptoms, lifestyle, mind body practices, patient-reported outcomes, psychosocial context, self-care behaviors, and survivorship needs.

However, this alignment between Digital Twins and Integrative Oncology is not automatic. As highlighted in the SWOT analysis, the same technologies that may support personalization and continuity of care also introduces risks related to data quality, fragmented documentation, algorithmic bias, privacy, autonomy, digital literacy, inequitable access, and excessive optimization of health behaviors. Digital Twins may support self-care, symptom management, prevention, and survivorship, but diagnosis, prognosis, and cancer treatment decisions should remain embedded in professional oversight and shared decision-making.

The priority for Integrative Oncology is therefore preparation. The field needs interoperable data infrastructures, standardized documentation of lifestyle and integrative interventions, consistent patient-reported outcome measures, digital competencies among health professionals, and ethical frameworks that protect patient choice and human-centered care. Without these foundations, Integrative Oncology risks remaining invisible in future AI-driven systems. With them, Digital Twins could become a catalyst for Whole Person Health, helping to connect conventional cancer treatment, evidence-informed integrative care, and patient-centered self-care across the cancer continuum.

## Data Availability

The original contributions presented in the study are included in the article/supplementary material. Further inquiries can be directed to the corresponding author.
